# Dimensions of Learning Organizations Questionnaire (DLOQ) in a low-resource health care setting in Nepal

**DOI:** 10.1186/1478-4505-13-6

**Published:** 2015-01-22

**Authors:** Mia Leufvén, Ravi Vitrakoti, Anna Bergström, Ashish KC, Mats Målqvist

**Affiliations:** International Maternal and Child Health (IMCH), Department of Women’s and Children’s Health, Uppsala University, SE-751 85 Uppsala, Sweden; Foundation for Maternal and Child Health, Maternity Hospital Road, Thapathali, Kathmandu, 44600 Nepal; UNICEF, Nepal Country Office, PO Box 1187, UN House, Pulchowk, Lalitpur, Kathmandu, Nepal

**Keywords:** Dimensions of the learning organization questionnaire, Health systems, Learning organizations, Nepal

## Abstract

**Background:**

Knowledge-based organizations, such as health care systems, need to be adaptive to change and able to facilitate uptake of new evidence. To be able to assess organizational capability to learn is therefore an important part of health systems strengthening. The aim of the present study is to assess context using the Dimensions of the Learning Organization Questionnaire (DLOQ) in a low-resource health setting in Nepal.

**Methods:**

DLOQ was translated and administered to 230 employees at all levels of the hospital. Data was analyzed using non-parametric tests.

**Results:**

The DLOQ was able to detect variations across employee’s perceptions of the organizational context. Nurses scored significantly lower than doctors on the dimension “Empowerment” while doctors scored lower than nurses on “Strategic leadership”. These results suggest that the hospital’s organization carries attributes of a centralized, hierarchical structure that might hinder a progress towards a learning organization.

**Conclusions:**

This study demonstrates that, despite the designing and developing of the DLOQ in the USA and its main utilization in company settings, it can be used and applied in hospital settings in low-income countries. The application of DLOQ provides valuable insights and understanding when designing and evaluating efforts for healthcare improvement.

## Background

Many low- and middle-income countries have health systems that are weak and fragile, lacking the capacity to put knowledge into practice and expand the delivery of health services to those in need [1]. At present, research on how to measure and identify health systems weaknesses and strengths is very scant. Without a thoroughly validated and scientifically sound concept or framework to assess the health systems policy-makers have little guidance on what they can and should strengthen [2].

In the field of human relations, research on organizational culture has been ongoing for decades. However, it is only in recent years that health researchers have become aware of the organizational culture as an important characteristic that may influence the effectiveness and/or success in implementing interventions in different health care settings. The research into knowledge-translation in health systems has increased during the last decade, particularly because of the recognition of its importance for achieving many of the Millennium Development Goals [3].

Today’s health care systems are busy, complex, and occasionally chaotic and under a constant demand from policy makers, health care analysts, and funders to deliver the best possible health care that is also operative and cost-efficient. To meet these increasing demands in a changeable environment is a challenging task, even in a high-income setting, and can be seen as an insuperable task in low- and middle-income settings, where health professionals and policy makers have to deal with constraints on human and financial resources, weak health systems, lack of infrastructure, and the need of behavioral change. Nevertheless, it is in these countries that the need to create functioning health systems that put “what works” into practice is particularly important, as ineffective treatments can drain limited resources resulting in further health inequities [4, 5].

In this ever-changing climate, the organizations that succeed are those who can continuously transform and adapt to the new circumstances, i.e., those who can adopt characteristics of a learning organization; this is equally true for health systems [6–8]. For the health care sector, in particular, the ability to learn is essential since knowledge and skills can rapidly become obsolete due to the continuous evolution in science and medicine [9]. This is crucial for both employee satisfaction and the overall quality of health care itself. The transformation of enterprises and organizations into learning organizations has been proposed as a key strategy for improving their effectiveness and efficiency. While the principle of learning organizations has been applied extensively in the corporate environment, it is a relatively new concept in health systems [10].

There are a multitude of definitions of what constitutes a learning organization, but there are also a few major convergent factors among them. Continuous learning and improvement have been put forward as important themes, and Garvin and Lewis propose the importance of creation, acquisition, and transfer of knowledge [11, 12]. Senge and Molainen mention individual, team, and organizational learning anchored in concrete values, visions, and goals, as well as change and transformation [8, 13]. Armstrong and Foley refer, in turn, to the appropriate processes and cultural and structural facets that support learning and development [14]. In line with these themes, there is a growing understanding that the dimensions of a learning organization encompass some basic elements of leadership, strategy, participative policymaking, continuous learning, dialogue and inquiry, team learning, empowerment, and facilitating processes and structures [11, 15–17].

Many difficulties regarding the assessment of organizational culture lie in the fact that there are an abundance of instruments for assessing and measuring them, applied to a large number of settings, each instrument with their own theoretical background. Moreover, these instruments have mainly been developed for, and tested in, high-income settings, resulting in a lack of well-established and/or validated instruments for low- and middle-income settings.

### The Dimensions of Learning Organization Questionnaire (DLOQ)

Moilanen identified and compared some of the instruments available in terms of scope, depth, and reliability [14]. They concluded that the Dimensions of the Learning Organization Questionnaire (DLOQ), developed by Marsick and Watkins [18], meets the three criteria of comprehensiveness, depth, and validity, and also integrates important attributes of the learning organization [19–22]. According to Marsick and Watkins [18], a learning organization has two components; the first represents the people who comprise an organization, and the second represents the structures and culture created by the social institution of the organization [18]. Furthermore, this model states that there are four levels of a learning organization: i) the individual level, which is composed of two dimensions of organizational learning, namely continuous learning and dialogue and inquiry; ii) the team or group level, which is reflected by team learning and collaboration; iii) the organizational level, which has two dimensions of organizational learning, namely embedded systems and empowerment; and iv) the global level, which consists of two dimensions of organizational learning, namely systems connection and strategic leadership. Each of these levels belongs to one of the two components mentioned earlier. This framework makes it clear that, in order to move towards the desired goal or outcome, an organization has to both work with people at the individual and group level, as well as create facilitative structures to support and capture learning [20, 23].

The DLOQ is designed to measure learning culture in organizations and intends to capture the employee’s perception regarding the seven dimensions in order to help the organization get a clearer picture on where they are versus where they need to be [18, 20]. The seven dimensions are of the positive nature and cultural aspects of a supportive learning organization, which encourages dynamic organizational learning processes (Table 1).

**Table 1:** **Definitions of constructs for the dimensions of the learning organization questionnaire**

Dimension	Definition
Create continuous learning opportunities	Learning is designed into work so that people can learn on the job; opportunities are provided for ongoing education and growth.
Promote inquiry and dialogue	People gain productive reasoning skills to express their views and the capacity to listen and inquire into the views of others; the culture is changed to support questioning, feedback, and experimentation.
Encourage collaboration and team learning	Work is designed to use groups to access different modes of thinking; groups are expected to learn together and work together; collaboration is valued by the culture and rewarded.
Create systems to capture and share learning	Both high- and low-technology systems to share learning are created and integrated with work; access is provided; systems are maintained.
Empower people toward a collective vision	People are involved in setting, owning, and implementing a joint vision; responsibility is distributed close to decision making so that people are motivated to learn toward what they are held accountable to do.
Connect the organization to its environment	People are helped to see the effect of their work on the entire enterprise; people scan the environment and use information to adjust work practices; the organization is linked to its communities.
Provide strategic leadership for learning	Leaders model, champion, and support learning: leadership uses learning strategically for business results.
Key results	
Financial performance	State of financial health and resources available.
Knowledge performance	Enhancement of products and services because of learning and knowledge capacity (lead indicators of intellectual capital).

(Table adapted from Marsick and Watkins, 2003 [18]).

There are currently two versions of the DLOQ, one full version with 43 measurement items, which has been shown to be useful as a diagnostic tool for practitioners who want a comprehensive assessment and information of the learning culture in order to make decisions on where to intervene [20]. The second version is an abbreviated form that contains 21 of the original 43 items but still possesses construct validity and reliability. This version is also better suited for scholars that want to use the DLOQ as a research instrument. There are three adequate measurement items for each of the seven dimensions included [20].

The aim of this study is to assess context using the DLOQ in a low-resource hospital setting in Nepal.

## Methods

### Setting

Since July 2012, an intervention trial to implement a simplified neonatal resuscitation protocol provided by Helping Babies Breathe (HBB) has been ongoing at Paropakar Maternity and Women’s Hospital in Kathmandu, Nepal [24], which is a central referral hospital of the country and also a teaching hospital for medical and nursing students on all levels. More than 500 medical interns are enrolled for internship each year and the nursing students from nearby colleges are placed at the hospital for 1 month of internship, where they rotate to new wards every week. It has a staff capacity of 627, of which 54 are doctors, 172 nurses, 40 paramedical, 82 administrative and finance, 247 support staff, and 27 others. The hospital has 415 beds, out of which 336 are allocated to indoor admission with 241 for obstetrics, 61 for gynecology, and 34 are for newborns; 79 are service beds. The delivery rate is close to 23,000 live births/year and the perinatal mortality rate is currently at 30/1,000 births. The majority of the deliveries take place in one of the three labor rooms [25, 26].

### Data collection

For this study, the shorter version of the DLOQ with 21 items was considered most appropriate because of its preferable psychometric properties, as well as its ease of completion and, thus, its reduction of loss to follow-up. The dimensions were measured on a 6-point Likert scale (1 – almost never, 6 – almost always; Table 2). The section pertaining to financial performance was omitted because the purpose of this study was only to capture a snapshot of this particular hospital’s organization and gain insights into learning-related strengths and weaknesses.

**Table 2:** **Descriptive statistics as per statement**

Statement	N	Mean	Median	Std. deviation	Range	Normality ***P***value
Dimension 1. Continuous learning						
Q1. In my organization, people help each other learn.	135	3.99	4	1.607	2–6	0.000
Q2. In my organization, people are given time to support learning.	134	3.44	3	1.539	2–6	0.000
Q3. In my organization, people are rewarded for learning.	131	2.36	2	1.525	2–6	0.000
Dimension 2. Dialogue and inquiry						
Q4. In my organization, people give open and honest feedback to each other.	135	3.14	3	1.589	2–6	0.000
Q5. In my organization, whenever people state their view, they also ask what others think.	134	3.19	3	1.436	2–6	0.000
Q6. In my organization, people spend time building trust with each other.	133	3.09	3	1.485	2–6	0.000
Dimension 3. Team learning and collaboration						
Q7. In my organization, teams/groups have the freedom to adapt their goals as needed.	134	3.19	3	1.606	2–6	0.000
Q8. In my organization, teams/groups revise their thinking as a result of group discussions or information collected.	133	3.25	3	1.549	2–6	0.000
Q9. In my organization, teams/groups are confident that the organization will act as their recommendations.	134	3.43	3	1.499	2–6	0.000
Dimension 4. Embedded systems						
Q10. My organization creates systems to measure gaps between current and expected performance.	134	3.10	3	1.491	2–6	0.000
Q11. My organization makes its lessons learned available to all employees.	132	3.28	3	1.608	2–6	0.000
Q12. My organization measures the results of the time and resources spent on training.	135	3.21	3	1.604	2–6	0.000
Dimension 5. Empowerment						
Q13. My organization recognizes people for taking initiatives.	132	2.87	3	1.714	2–6	0.000
Q14. My organization gives people control over the resources they need to accomplish their work.	131	3.21	3	1.502	2–6	0.000
Q15. My organization supports employees who take calculated risks.	133	3.22	3	1.583	2–6	0.000
Dimension 6. Systems connections						
Q16. My organization encourages people to think from a global perspective.	132	3.01	3	1.454	2–6	0.000
Q17. My organization works together with the outside community to meet mutual needs.	135	3.59	4	1.604	2–6	0.000
Q18. My organization encourages people to get answers from across the organization when solving problems.	133	3.02	3	1.459	2–6	0.000
Dimension 7. Strategic leadership						
Q19. In my organization, leaders mentor and coach those they lead.	134	3.83	4	1.597	2–6	0.000
Q20. In my organization, leaders continually look for opportunities to learn.	135	3.79	4	1.579	2–6	0.000
Q21. In my organization, leaders ensure that the organization’s actions are consistent with its values.	134	3.61	3	1.501	2–6	0.000

*P* >0.05, normally distributed data; *P* <0.05, non-normally distributed data.

A pilot testing of the modified DLOQ showed that the hospital personnel’s knowledge of the English language was not sufficient for a proper understanding of the statements. Translation to Nepali and back-translation to English was performed by two independent translators. The back-translation was then assessed in terms of conceptual equivalence, clarity and language, and cultural adequacy by the first author. After the translation of the 21 items was conducted, these were transferred into a form with additional statements regarding the respondent’s profession and primary workplace.

All nurses, nursing students, medical interns, medical officers, pediatricians, gynecologists, and obstetricians that worked at, or did their internship at, the hospital during a period of 9 weeks from November 2012 to January 2013 were invited to fill out the DLOQ. The form was distributed to the staff to fill out anonymously and was later collected either by the matron in charge of the ward or members of the research team.

### Data analysis

The collected data was subjected to quantitative, descriptive analysis using SPSS (version 12.0). Due to the relatively small data sample, a test for normality was performed, using the Shapiro-Wilks test. Alpha level was set to 0.05, which led to the rejection of the null hypothesis for all the statements as well as all the dimensions. Non-parametric tests were therefore used for the further analysis of the data. The Kruskal-Wallis test was used to test for significant differences between the different groups of professionals. Mann-Whitney tests were used to compare groups that showed a significant difference through the Kruskal-Wallis test.

## Results

Out of the 230 forms distributed, 135 were collected, corresponding to a response rate of 59%. Out of the respondents, 36% (49) were nurses, 28% (38) were nursing students, and 26% (35) were doctors, whereof 6 were medical interns, and an additional 10% (13) of the respondents had failed to answer the question regarding profession. The survey thus covered 54% (29/54) of the hospital’s doctors and 28% (49/172) of the nurses.

The descriptive statistics for the statements and the proposed dimensions are displayed in Tables 2 and 3. The means of Q1 to Q21 ranges between 2.36 on Q3 (“In my organization, people are rewarded for learning”) to 3.99 on Q1 (“In my organization, people help each other learn”), the mean standard deviation for the statements, as calculated from the table, is 1.549. In Table 3, the means of the dimensions, calculated by adding all individual scores for each item, ranges between 3.09 on the dimension measuring empowerment (5) to 3.79 on the dimension measuring strategic leadership (7). The mean standard deviation for the dimensions, as calculated from the table, is 1.211. Statement 1 got the highest overall score and reads as follows: “In my organization people help each other learn”. The lowest score was noted for statement 3, which reads as follows: “In my organization people are rewarded for learning”.

**Table 3:** **Descriptive statistics as per dimension**

Dimension	Mean	Median	Range	Std. deviation	Normality ***P***value
Continuous learning (1)	3.24	3	2–6	1.120	0.002
Inquiry and dialogue (2)	3.14	3	2–6	1.116	0.010
Team learning (3)	3.29	3	2–6	1.207	0.022
Embedded systems (4)	3.17	3	2–6	1.203	0.014
Empowerment (5)	3.09	3	2–6	1.327	0.001
Systems connection (6)	3.21	3	2–6	1.187	0.002
Strategic leadership (7)	3.75	4	2–6	1.318	0.007

*P* >0.05, normally distributed data; *P* <0.05, non-normally distributed data.

**Figure 1:**
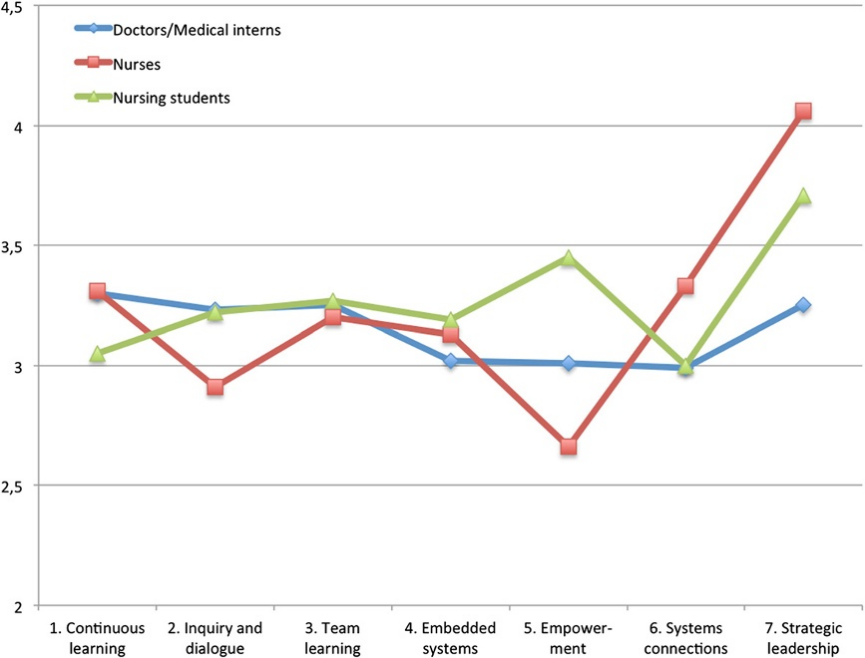
**Diagram showing the distribution of the means for each profession as per dimension.**

Scores for the dimensions distributed by profession are displayed in Figure 1. Doctors and medical interns were grouped together since they share a common work situation. Nursing students are only at the hospital for one month and have a teaching position and were thus treated as a separate group from nurses. For doctors/medical interns, the mean ranged from 2.99 on the dimension measuring systems connection (6) to 3.30 on the dimension measuring continuous learning (1), with a mean standard deviation, as calculated from the table, of 0.778. For the nurses the mean ranged from 2.66 on the dimension measuring empowerment (5) to 4.06 on the dimension measuring strategic leadership (7), with a mean standard deviation of 0.559. The nursing students’ mean ranged from a 3.00 on the dimension measuring systems connection (6) to a 3.71 on the dimension measuring strategic leadership (7) and a mean standard deviation of 1.488. The Kruskal-Wallis test indicated a significant difference between the means of the different professions regarding empowerment and strategic leadership (dimensions 5 and 7; Table 4). In the next step, Mann-Whitney tests comparing the relation between each of the professions in dimensions 5 and 7 were performed. A significant difference (*P* = 0.005) between nurses and doctors/medical interns regarding the dimensions measuring strategic leadership (dimension 7) was noted. Between nurses and nursing students there was a significant difference (*P* = 0.042) in the dimension measuring empowerment (dimension 5). Between the nursing students and the doctors/medical interns there were no significant differences found in dimensions 5 and 7.

**Table 4:** **Descriptive statistics as per profession and dimension**

Profession	Dimension	1	2	3	4	5	6	7
Doctors/medical interns	N	35	35	35	35	34	34	35
	Mean	3.30	3.23	3.25	3.02	3.01	2.99	3.25
	Std. Deviation	0.654	0.707	0.658	0.824	0.847	0.855	0.901
Nurses	N	46	47	48	46	44	49	48
	Mean	3.31	2.91	3.20	3.13	2.66	3.33	4.06
	Std. Deviation	1.187	1.156	1.308	1.332	1.277	1.226	1.413
Nursing students	N	37	38	37	37	38	35	37
	Mean	3.05	3.22	3.27	3.19	3.45	3.00	3.71
	Std. Deviation	1.437	1.400	1.537	1.439	1.679	1.374	1.550
Missing profession	N	12	12	12	13	11	12	13
	Mean	3.39	3.56	3.86	3.67	3.85	3.92	4.10
	Std. Deviation	0.827	0.796	0.758	0.733	0.656	1.026	0.725
	Kruskal-Wallis	0.639	0.100	0.171	0.291	0.008*	0.068	0.030*
	*P* value							

*P <0.05, significant.

## Discussion

Results indicate that the respondents scored lowest on empowerment, with an overall score of 3.09 out of 6, indicating a potential area for improvement. The definition of this dimension, as proposed by Marsick and Watkins [18], is “*People are involved in setting, owning, and implementing a joint vision; responsibility is distributed close to decision making so that people are motivated to learn toward what they are held accountable to do*”. For an organization that strives toward becoming a learning organization this is a concern due to the importance placed on teamwork and empowerment in the management literature and in learning organization models [27–30]. In earlier studies using the DLOQ in different company settings, a low score on the empowerment dimension has been connected with organizations that display a centralized hierarchical structure, where the lower level employees may have limited access to information and limited authority to make decisions, which leaves little or no incentive to take initiative for learning or incorporation of new ideas [31–35].

The predominant organizational structure for hospitals is, by tradition, mostly bureaucratic, governed by hierarchical structures, rigid rules, and standard procedures and processes, which leave a narrow aperture for employee participation in decision-making [36]. The lower-level employees in particular, in the case of hospitals, the nurses, and students, have little or no influence. Decision-making is centralized and the employees are expected to implement decisions that they have not been involved in making [37]. Although this traditional structure is increasingly challenged and transformed into a more non-hierarchical, decentralized, and team-orientated organization, there are still many hospitals, especially in developing countries such as Nepal, which continue with the traditional hospital management structure. Partly because of tradition and culture but also because of a lack of funding and knowledge on how to transform themselves.

The dimension that scored the overall highest score was the one concerning strategic leadership (3.75), which is defined as follows: “Leaders model, champion, and support learning: leadership uses learning strategically for business results”. This can be attributed to the high regard employees bestow upon their leaders in a high power distance society such as the hospital setting. There may also be an unwillingness to critique managers and leaders due to the hierarchical structure.

When we take a closer look at how the different professions scored on the dimensions we see that the nurses, in this case considered to be low-level employees, scored strategic leadership the highest, with 4.07, and scored empowerment the lowest, with 2.66, whereas the doctors and medical interns scored continuous learning the highest, with 3.30, and systems connection the lowest, with 2.99. Interestingly, the nursing students, whom one could expect to score similar to the nurses because of their supposed low position in the hierarchy, actually scored systems connection the lowest (3.00) and had empowerment as their second highest dimension (3.45) after strategic leadership (3.71). A possible explanation to this result could be that the nursing students are only present at the hospital one month, and during that month they rotate between workplaces. This gives them a unique perspective of the organization, especially regarding how well it connects with its outer and inner environment, and therefore their perception of the dimension measuring systems connection and empowerment differ from that of the other professions. These findings concur with the earlier reasoning regarding how a hierarchical structure can influence an employee’s perception of a learning organization.

Learning at the group and organizational levels depends mostly on a positive propensity to teamwork and good communication between the members of the organization. What becomes obvious when reviewing the literature on learning organizations and the DLOQ is that its various dimensions need to be considered simultaneously and in an integrated manner. Systems theory conceives of learning organizations as comprising inter-dependent building blocks at the individual, group, organizational, and global level. The idea is that the dimensions and propensities detected at various levels necessarily combine, interact, and co-evolve to shape the disciplines of an advanced learning organization [38]. The main implication here is that the visible progress detected in one or more dimensions needs to be complemented with equal progress in other dimensions to foster a complete effective learning cycle and obtain the overall capabilities of an advanced learning system [33].

The results of this exploratory study provide some evidence of how perceptions vary across organizational levels at the Paropakar Maternity and Women’s Hospital concerning the learning organization. However, the results should be viewed taking into consideration the study’s limitations, which include the limited sample size of 135 collected forms with a response rate of 59% coupled with the uncertainty concerning the missing forms. There is also the issue of the people who potentially could have answered the form but did not, which of course also adds a level of uncertainty regarding the results. No background data, other than profession, was collected. This hampered the possibility to further evaluate the results as well as to analyze missing data.

For this study, the DLOQ form underwent translation from English to Nepali. Due to the time limitations it was not possible to perform an extensive translation process using professional translators and to thoroughly assess the forward and backward translations for conceptual equivalence, clarity, language, and cultural adequacy. This poses an additional limitation to this study, as there is a likelihood that the translation in some parts fails to capture the conceptual meaning of the items possibly due to language or cultural misinterpretation.

It should also be noted that the 13 respondents who did not state their profession scored higher on average in all dimensions. It can only be speculated on what the reasons for this could be. One reason could be “willingness-to-please” in combination with fear of being identified. Thus, the answers would not reflect the real situation, but pull all scores upwards. Omitting the respondent with missing data about profession did not, however, change the results of the Kruskal-Wallis test. In an earlier study, Sinha identified submissiveness and fear of independent decision-making as some of the generic attributes of Indians [39]. This observation was further developed by Awasthy and Gupta [34], where they propose that other countries with a culture similar to that of India, for example, other countries in South Asia and perhaps some countries in rest of Asia, may exhibit similar phenomena in relation to the learning organization [39]. More research would be needed to test this conjecture. In another study using the DLOQ in the Taiwanese context [32], the authors found that Taiwanese and Chinese employees may have interpretations on the dimensions that differ from those held by their American counterparts. This, of course, raises the statement of cultural context and understanding of the DLOQ as a possible limitation for this study, especially when considering the above-mentioned limitations with the translation. There may be specific socio-cultural attributes in the Nepali context that affect how the respondents interpret the items in the questionnaire. The prominent hierarchical structure may produce a culture of “willingness-to-please” in the organization that leads the respondents to not score accordingly to their actual perception, but to what they believe the researcher and/or administration wants them to answer. This could be exaggerated if the respondents have confidentiality concerns and fear retribution if they do not score accordingly. This would challenge the credibility of the results as well as the test itself and might render the suggested findings invalid.

Elaborating on the possible socio-cultural limitations for this questionnaire, there is also a limitation in the fact that this instrument is developed in a western, high-income context, and thus there might be dimensions present that this tool does not measure. For example, the influence of limited economical resources and corruption, which in many countries are important factors affecting organizations.

Further limitations include biases regarding personal attitudes, job satisfaction, tenure with the organization, and the effects of common method variance.

## Conclusions

The findings from this study provide useful information for the hospital administration regarding the areas where there is a need for improvement. It suggests that this hospital’s organization carries attributes of a centralized, hierarchical structure that might hinder a progress towards a learning organization. A way of addressing this could be to improve the communication in the organization, which would increase knowledge and participation. The involvement of the lower level employees and the provision of resources and time for learning would contribute to further empowerment of the workers and a progression towards a learning organization.

This study demonstrates that, despite the design and development of the DLOQ in the USA and its main utilization in company settings, it can be used and applied in hospital settings. The results also contribute with useful information for the HBB intervention as they provide a clearer picture of the conditions of the setting in which the researchers are implementing the intervention. These conditions might ultimately influence the reception, implementation, and outcome of this intervention. Because this study took place during the baseline and training part of the HBB intervention, this study provides a snapshot of the hospitals organization before the intervention and therefore serves as a base for investigation of how this intervention affected the hospitals organization.

Finally, this instrument can be used in conjunction with other validated measurement tools to expand and further research the realm of cultural factors which may impact organizational development. Future studies should investigate the relationships between organizational learning and other cultural factors such as the effects of trust, ethics, and justice on the boost of organizational learning.

## Abbreviations

DLOQ: Dimensions of the learning organization questionnaire
HBB: Helping babies breathe.
